# Inflammatory response to chronic nicotine-containing electronic cigarette exposure in a rat model of myocardial infarction

**DOI:** 10.18332/tid/204010

**Published:** 2025-05-31

**Authors:** Jianru Shi, Wangde Dai, Juan Carreno, Jaspreet Sachdeva, Jesus Chavez, Michael T. Kleinman, David A. Herman, Rebecca J. Arechavala, Irene Hasen, Amanda Ting, Robert A. Kloner

**Affiliations:** 1 Cardiovascular Research Institute, Huntington Medical Research Institutes, Pasadena, United States; 2 Division of Cardiovascular Medicine, Keck School of Medicine, University of Southern California, Los Angeles, United States; 3 Department of Environmental and Occupational Health, University of California Irvine, Irvine, United States

**Keywords:** e-cigarette, lung inflammation, cardiac inflammatory genes, blood parameters

## Abstract

**INTRODUCTION:**

The long-term effects of chronic electronic cigarette (e-cigarette) exposure on lung and heart inflammation during the healing phase of myocardial infarction (MI) remain unexplored. Additionally, the impact of e-cigarette exposure on blood parameters in this context is unclear. This study aims to assess e-cigarette with nicotine (e-cig Nic+) effects on lung histology, inflammatory gene expression in cardiac tissue, and blood parameters during MI recovery.

**METHODS:**

Sprague Dawley rats of both sexes underwent proximal left coronary artery occlusion to induce a large anterior wall MI. After one week, rats were randomized to either air or e-cig Nic+ exposure for 12 weeks.

**RESULTS:**

In the lungs, e-cig Nic+ exposure led to a significant accumulation of inflammatory cells within the alveolar spaces and increased inflammatory cell numbers in the lung parenchyma compared to the air group. Numerically elevated levels of malondialdehyde (MDA), an oxidative stress biomarker, were observed in the e-cig Nic+ group. In the heart, a PCR array analysis of inflammatory cytokines and receptors revealed that 70 out of 84 inflammatory-related genes were downregulated in the e-cig Nic+ group, with 11 reaching statistical significance. Additionally, the blood of rats exposed to e-cig Nic+ exhibited significantly lower white blood cell, lymphocyte, and platelet counts compared to the air group.

**CONCLUSIONS:**

Chronic exposure to e-cig Nic+ exacerbates lung inflammation, alters inflammatory gene expression in the heart, and suppresses immune cell counts in the blood during MI recovery. These findings suggest that e-cigarette with nicotine aerosol inhalation contributes to lung lesions and dampens immune and inflammatory responses in an already compromised MI setting.

## INTRODUCTION

Since their invention in 2003, electronic cigarettes (e-cigarettes) have surged in popularity, particularly among younger populations, as an alternative to traditional tobacco products. This shift has been driven by factors such as the perception of e-cigarettes as a safer option, the wide variety of flavors available, and aggressive marketing strategies targeting youth. Recent studies have highlighted the evolving landscape of e-cigarette usage, particularly among adolescents and young adults. As of 2023, e-cigarettes remain the most commonly used tobacco product among US youths, with 10.0% of high school students and 4.6% of middle school students reporting current use^1^. Interestingly, there has been a notable decline in e-cigarette usage among high school students, down from 14.1% in 2022 to 10.0% in 2023 ^1^. This decrease is likely influenced by comprehensive tobacco control measures and increased awareness of health risks. However, despite this decline, a significant proportion of current e-cigarette users (nearly 40% of high school users) engage in frequent use, underscoring the ongoing public health concern^1^.

E-cigarette liquids contain substances like propylene glycol, vegetable glycerin, glycerol, and acrolein – compounds known to generate reactive oxygen species (free radicals) that can damage cell membranes and trigger inflammation^2^. The electronic components of e-cigarettes heat these liquids and nicotine to high temperatures, creating ultrafine particles that enter the lungs and potentially the blood stream more extensively than conventional tobacco smoke^3^. E-cigarettes are often used to deliver a solution of nicotine, a substance that causes vasoconstriction, stimulates the sympathetic nervous system, and is highly addictive. This has led to nicotine addiction in teenagers, potentially resulting in behavioral issues such as anxiety, mood disorders, and cognitive impairment^4^.

Although e-cigarette vapor is widely perceived as less harmful than traditional smoking, its effects on the cardiovascular system are not entirely explored, especially in the setting of ongoing cardiac pathology, such as healing myocardial infarction. With relapse rates exceeding 60% after myocardial infarction (MI)^5^, some might assume that e-cigarettes are a safer alternative that would not exacerbate left ventricular (LV) remodeling post-MI. However, there are no data addressing the impact of e-cigarette exposure during the critical healing phase of MI.

In the present study, we investigated whether exposure to e-cigarette vapor containing nicotine induces pulmonary pathology, alters cardiac inflammatory gene expression, and affects blood parameters during the MI healing phase in a rat experimental model.

## METHODS

This study received approval from the Institutional Animal Care and Use Committees (IACUC) at both Huntington Medical Research Institutes and the University of California, Irvine. Both institutions hold accreditation from the Association for Assessment and Accreditation of Laboratory Animal Care International (AAALAC). All experimental procedures were conducted in strict compliance with the Guidelines for the Care and Use of Laboratory Animals, as outlined in NIH Publication No. 85-23 (National Academy Press, Washington, D.C., revised 2011). Our study was conducted in compliance with the ARRIVE (Animal Research: Reporting of In Vivo Experiments) guidelines to ensure rigorous and reproducible animal research (https://arriveguidelines.org).

### Induction of myocardial infarction in rats

Myocardial infarction (MI) was induced in 10-week-old Sprague Dawley rats of both sexes through permanent ligation of the proximal left coronary artery. The procedure was conducted under anesthesia using an intraperitoneal injection of ketamine (90 mg/kg) and xylazine (10 mg/kg). A left thoracotomy was performed to access the heart, after which the pericardium was carefully incised. The proximal left coronary artery was identified, encircled with a 4–0 silk suture, and then permanently ligated. Successful occlusion was confirmed by the development of cyanosis and akinesis in the anterior wall of the left ventricle, indicating effective myocardial ischemia. Following the procedure, the thoracic incision was meticulously closed in layers to ensure proper wound healing. The rats were then allowed to recover from anesthesia and were closely monitored before being returned to a clean cage with appropriate post-operative care.

### Nose-only exposure to e-cigarette vapor and grouping

One week after inducing MI, rats were subjected to a nose-only exposure system at the Air Pollution Health Effects Laboratory at the University of California, Irvine, following previously established methods^6^. Briefly, each rat was placed in an individual restraining tube designed to secure its body while allowing only the snout to be exposed to the test atmosphere. This setup, utilizing an exposure manifold (In-Tox Products, LLC, Clinton, MS), was specifically designed to minimize dermal exposure and ensure controlled inhalation of the test substance. The experimental groups included rats exposed to either e-cigarette vapor containing nicotine (e-cig Nic+, n=32) or filtered air (n=37) as a control. These exposures were conducted for 5 hours per day, 4 days per week, over a total duration of 12 weeks.

E-cigarette vapor was generated using an electronically controlled smoking machine, which was programmed with various puff regimens to simulate real-world vaping conditions. The generated aerosols exhibited particle size distributions comparable to those observed during human e-cigarette use. The e-liquid formulation contained 15 mg/mL of nicotine (L-Nicotine, Acros Organics, Lot: A0382410) dissolved in a 50:50 (vol/vol) mixture of propylene glycol (PG) and vegetable glycerin (VG), with an added tobacco flavoring (sourced from www.VaporFi.com). The selected nicotine concentration of 15 mg/mL was chosen to match the nicotine levels used in the National Institute on Drug Abuse (NIDA) Standard Research E-Cigarette (SREC), a reference product commonly employed in human exposure studies. This study design aimed to replicate human exposure patterns and investigate the potential physiological effects of chronic e-cigarette vapor inhalation following MI.

### Tissue collection for histological analysis

Following 12 weeks of exposure, the rats were transported from the Air Pollution Health Effects Laboratory at the University of California, Irvine, to Huntington Medical Research Institutes in Pasadena, CA. Upon arrival, the animals were given a period of 2 to 4 days to acclimate to their new environment before undergoing tissue collection. To ensure proper fixation and preservation of tissue structures, the rats were deeply anesthetized prior to organ extraction. Once a surgical plane of anesthesia was achieved, the lungs were carefully excised and perfused with 10% formalin via the trachea. This perfusion method was employed to prevent alveolar collapse and maintain lung architecture for subsequent histological examination. Following lung perfusion, both the heart and lungs were immersed in 10% formalin for additional fixation. Once adequately preserved, the organs were dissected and sectioned for further processing. Tissue sections (5 μm thickness) were stained using hematoxylin and eosin (H&E) to facilitate histological evaluation under a microscope.

### Malondialdehyde (MDA) assay for lipid peroxidation in serum samples

After blood collected from the right jugular vein under anesthesia, the samples were transferred into plain tubes without anticoagulants and allowed to clot at room temperature for 30 min. The samples were then centrifuged at 2500 rpm for 15 min at 4°C to separate the serum. The supernatant serum was carefully collected and stored at -80°C until further analysis. To evaluate oxidative stress levels, lipid peroxidation was measured using an MDA assay kit (Abcam, ab118970) according to the manufacturer’s instructions. This assay quantifies MDA, a byproduct of lipid peroxidation, as an indicator of oxidative damage. Serum samples (20 μL) were processed by adding 500 μL of 42 mM sulfuric acid (H_2_SO_4_) and 125 μL of phosphotungstic acid solution. The mixture was incubated, followed by centrifugation to obtain a pellet. The pellet was then resuspended in 200 μL of double-distilled water (ddH_2_O). To initiate the reaction, a thiobarbituric acid (TBA) solution was added to each sample and standard. The reaction mixture was incubated at 95°C for 60 min and subsequently cooled on ice for 10 min to stabilize the reaction products. Absorbance was measured at 532 nm using a BioTek Synergy H1 microplate reader. This method provided quantitative data on lipid peroxidation levels, allowing for the assessment of oxidative stress in serum samples.

### Blood sample collection and analysis

After 12 weeks of exposure, 0.5 mL of blood was collected from each rat right jugular vein under anesthesia. The collected blood was transferred into EDTA-coated tubes to prevent coagulation. The samples were gently mixed by inversion and were then processed for cell count analysis, including white blood cell (WBC), lymphocyte, and platelet counts, using an automated IDEXX LaserCyte Dx Hematology Analyzer.

### PCR gene expression analysis in heart tissue

To assess gene expression changes in heart tissue, a PCR gene array targeting inflammatory cytokines and receptors was performed in both the e-cigarette and air exposure group (n=4 female rats in each group). Total RNA was extracted from the heart tissue using the Trizol reagent (Invitrogen) following the manufacturer’s instructions. To ensure the removal of genomic DNA contamination, the isolated RNA was treated with RNase-free DNase and subsequently purified using the RNase Mini Kit (Qiagen). The quality and concentration of RNA were assessed before proceeding with the reverse transcription process.

For cDNA synthesis, 500 ng of purified total RNA was used in a reverse transcription reaction utilizing the RT2 First Strand Kit (Qiagen). The synthesized cDNA was then subjected to real-time quantitative PCR analysis using the Rat Inflammatory Cytokines & Receptors PCR Array (PARN-011Z, Qiagen). This array was designed to profile the expression of key genes involved in the inflammatory response. The amplification and detection process were carried out using the Bio-Rad CFX96 Touch Real-Time PCR Detection System. This approach enabled the quantification of inflammatory mediators, providing insights into the molecular mechanisms underlying inflammation in the heart following experimental conditions.

### Statistical analysis

All data are presented as mean ± standard error of the mean (SEM). Statistical analyses were performed using two-tailed Student’s t-test to compare group differences. A p<0.05 was considered statistically significant, indicating meaningful differences between experimental conditions.

## RESULTS

The rats utilized in this study were also included in previous investigations examining the effects of e-cigarette exposure on left ventricular (LV) remodeling and cardiac function. The findings from those analyses have been previously published^6^. However, the data presented in the current study are distinct and have not yet been reported in any full-length peer-reviewed publications. This study explores a distinct aspect of the research, offering new insights into the inflammatory response triggered by e-cigarette exposure during the post-MI healing phase. While the animals were part of multiple analyses, the findings discussed here represent an independent and previously unpublished evaluation of the collected data.

### E-cigarette exposure increased lung inflammation compared to air

**Figure 1:**
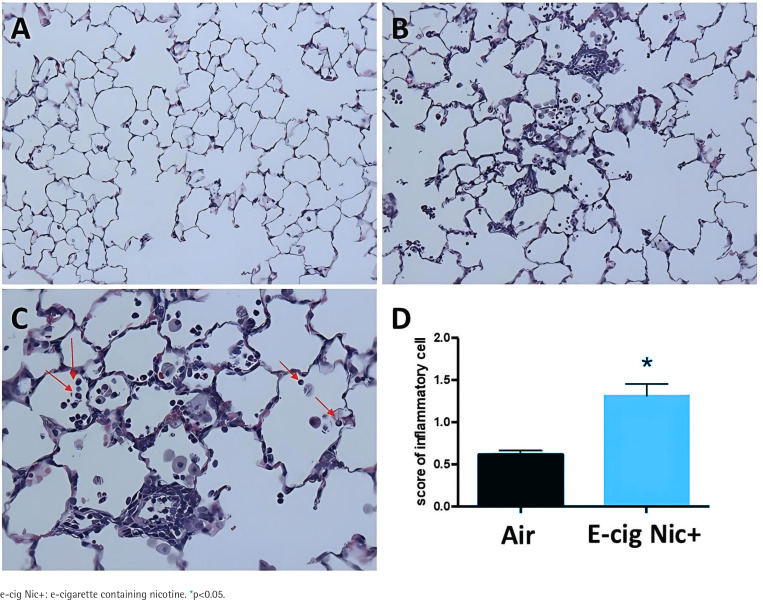
Representative H&E staining of lung sections: A) lung alveoli appeared normal in the air group; B and C) alveolar region of e-cig Nic+ exposed rats show a mark accumulation of inflammatory cells including neutrophil infiltration (red arrows); D) scores (0-4; where 0 = no inflammation and 4 = severe inflammation) of inflammatory cells in the lung parenchyma were significantly greater in the rats exposed to e-cig Nic+ (n=27) compared to the air group (n=33)

After 12 weeks of exposure, lungs were harvested, fixed in formalin under constant pressure, and sectioned into 5 μm slices. These sections were stained and prepared for examination via light microscopy. H&E staining revealed normal alveolar architecture in the air-exposed group (Figure 1A). In contrast, rats exposed to e-cig Nic+ showed a notable accumulation of inflammatory cells, including neutrophil infiltration in the alveolar region (Figure 1B and Figure 1C). Inflammatory cell scoring (0–4) indicated a significant increase in lung parenchymal inflammation in the e-cig Nic+ group (n=27) compared to the air group (n=33, p<0.05, t-test, Figure 1D).

### Serum levels of malondialdehyde (MDA) increased in e-cigarette groups compared to air

**Figure 2:**
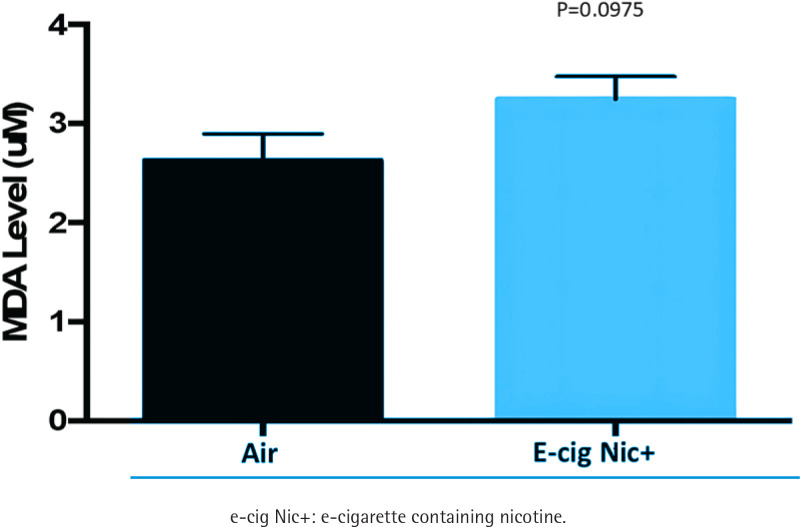
Serum levels of malondialdehyde showed a trend toward a numerical increase in e-cig Nic+ group (n=9) compared to the air group (n=9)

MDA is a biomarker of oxidative stress-induced lipid peroxidation. Serum MDA levels exhibited a nonsignificant trend toward an increase in e-cigarette groups (3.24 ± 0.23 μM, n=9) compared to the air group (2.63 ± 0.27 μM, n=9; p=0.0975) (Figure 2).

### E-cigarette exposure decreased WBC, lymphocytes, platelets in blood compared to air

**Figure 3:**
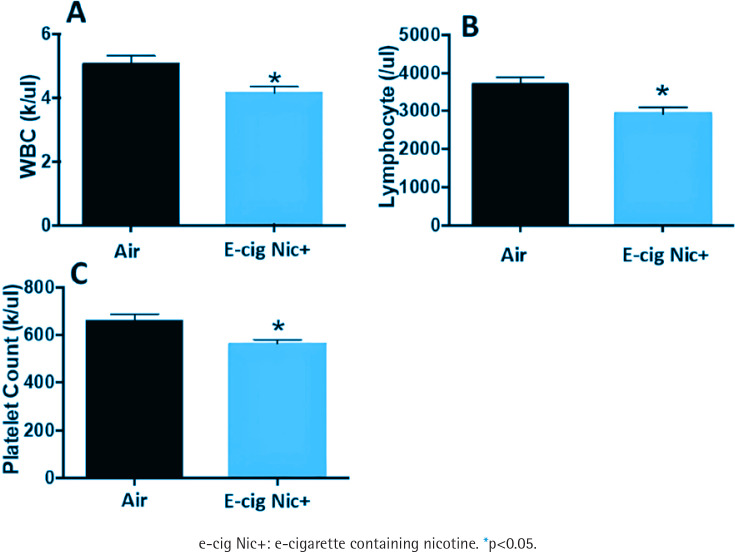
Cell counting analysis of blood samples revealed a significant decrease in: A) white blood cell count, B) lymphocyte count, and C) platelet count, in the e-cig Nic+ group (n=32) compared to the air group (n=35)

The blood tests showed that e-cig Nic+ group had a significant decrease in the number of white blood cells (WBC) and in lymphocytes. Furthermore, a significant decrease in platelet count was detected in e-cig Nic+ group (n=32) compared to the air group (n=35) (Figure 3).

### E-cigarette exposure downregulated 70 inflammatory genes expression in heart compared to air

**Figure 4:**
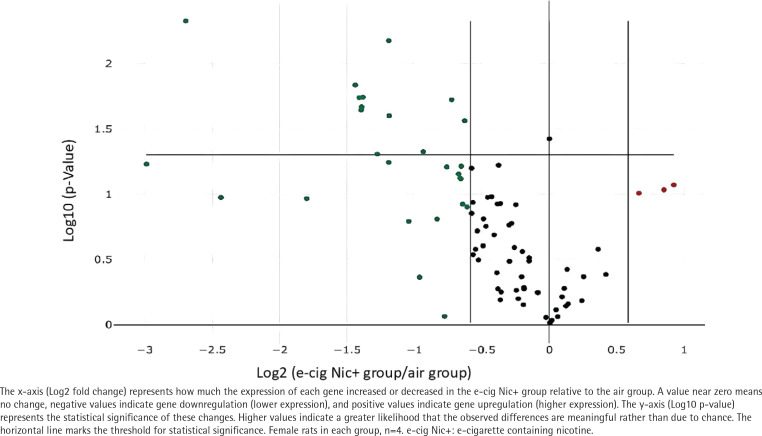
The RT-PCR (reverse transcription PCR) array volcano plot illustrates changes in the expression of inflammation-related genes in the e-cig Nic+ group compared to the air group; 70 out of 84 inflammatory-related genes were downregulated in the e-cig Nic+ group versus the air group, and 11 downregulated genes reached a significant difference assessed by the PCR array

After 12 weeks of exposure, a PCR array analysis was conducted to assess inflammatory cytokines and receptor gene expression in rat hearts (n=4 female rats in each group). The results revealed that 70 out of 84 inflammation-related genes were downregulated in the e-cig Nic+ group compared to the air-exposed group. Among these, 11 genes demonstrated substantial downregulation, with statistically significant differences, as illustrated in the Volcano plot (Figure 4). Specifically, the gene expressions of Interleukin 6 (IL6), Tumor Necrosis Factor Superfamily (TNF), Chemokine (C-C Motif) Ligand 12 (Ccl12), and C-X-C Motif Chemokine Receptor 3 (Cxcr3) were significantly reduced by -6.49 fold (p=0.0048), -2.63 fold (p=0.0227), -2.71 fold (p=0.0146), and -2.28 fold (p=0.0067), respectively, in the e-cig Nic+ group versus the air group (Table 1).

**Table 1:** Inflammatory cytokines and receptors genes with significant decrease in e-cig Nic+ versus air group

*Genes*	*Fold change*	p*
**Chemokines**		
Ccl12	-2.71	0.002
Ccl17	-2.61	0.018
Ccl24	-2.28	0.025
Ccl3	-2.66	0.018
Ccl5	-1.55	0.027
Cd40lg	-1.91	0.047
Cxcr3	-2.28	0.007
**Interleukins**		
Il1b	-6.49	0.005
**TNF superfamily**		
Faslg	-1.65	0.019
Tnf	-2.63	0.023
Tnfsf14	-2.63	0.021

e-cig Nic+: e-cigarette containing nicotine. Female rats in each group, n=4.

* Statistically significant at p<0.05.

## DISCUSSION

The key finding of this study is that exposure to e-cig Nic+ led to increased inflammatory cell infiltration in the lungs and elevated oxidative stress biomarkers in the serum. Additionally, e-cig Nic+ exposure downregulated the expression of inflammatory genes in cardiac tissue and reduced blood immune cell counts during the healing phase of myocardial infarction.

E-cigarette vapor is toxic to human alveolar macrophages, inducing excessive reactive oxygen species, inflammation, apoptosis, necrosis, and impaired bacterial clearance, suggesting potential lung immune dysfunction^7^. Impact of e-cigarette vapor on respiratory health remains a topic of ongoing research, and growing evidence suggests that exposure to e-cigarette aerosol elicits inflammatory responses in the lungs and promotes oxidative stress^8,9^. For instance, Garcia-Arcos et al.^10^ found that mice exposed to e-cigarette aerosols showed increased infiltration of inflammatory cells into the lungs, with macrophages being the most abundant cell type. Their analysis of tissue inflammation revealed higher numbers of macrophages in the lung tissue of mice exposed to nicotine-containing e-cigarette fluids, while the levels of neutrophils and lymphocytes remained unaffected. Schweitzer et al.^11^ found that even brief exposure to e-cigarettes rapidly triggers pulmonary responses, including neutrophil-driven lung inflammation, a swift increase in polymorphonuclear cells in the bronchoalveolar lavage fluid, and compromised lung endothelial barrier function. Our present study aligns with these findings, demonstrating that exposure to e-cig Nic+ during the MI healing phase results in the accumulation of inflammatory cells, primarily macrophages with a smaller presence of neutrophils, in the lungs. These observations underscore the detrimental effects of e-cigarette use on lung health. The accumulation of inflammatory cells in the lungs suggests that e-cigarette exposure exacerbates inflammatory responses. This highlights the need for further research to fully understand the long-term health implications of e-cigarette use and to inform public health policies aimed at mitigating these adverse effects.

E-cigarette use has been linked to oxidative stress and alterations in hematological parameters. Investigations on the impact of e-cigarette exposure on serum levels of malondialdehyde (MDA), a biomarker of oxidative stress, have demonstrated that MDA levels are significantly increased, indicating heightened oxidative stress. Suryadinata et al.^12^ reported that exposure to e-cigarette smoke increases free radicals in the blood, leading to reduced antioxidant superoxide dismutase levels and elevated malondialdehyde levels, which contribute to oxidative stress, airway damage, and inflammation. Consistent with this finding, our present study shows that e-cig Nic+ exposure during the MI healing phase resulted in increased lipid peroxidation in the serum after 12 weeks of exposure. Snoderly et al.^13^ examined the short-term effects of e-cigarette exposure in female BALB/cJ mice. Mice aged 12–16 weeks were randomly assigned to room air or e-cigarette exposure for three consecutive days. Blood and bronchoalveolar lavage fluid (BALF) were collected post-exposure for immune cell analysis. Results showed an increase in neutrophils in both blood (1.1–1.9-fold) and BALF (1.1–2.5-fold), along with a threefold rise in lymphocyte levels in BALF at 24 hours post-exposure, though these changes were not statistically significant. Histological analysis revealed that e-cigarette exposure led to increased neutrophil accumulation in the pulmonary microvasculature and heightened neutrophil-platelet interactions, potentially contributing to lung inflammation. These effects persisted for up to 48 hours of post-exposure. Corriden et al.^14^ conducted a study in which female C57BL/6 mice were exposed to e-cigarette vapor containing 24 mg/mL nicotine for four weeks before being subjected to an abdominal infection with *Pseudomonas aeruginosa*. The aim was to evaluate the impact of e-cigarette vapor on neutrophil function in host defense. Although total circulating white blood cell counts were not measured, the study found that fewer neutrophils migrated from the bloodstream to the infected peritoneal cavity in e-cigarette-exposed mice compared to air-exposed controls. This finding suggests that e-cigarette use may impair immune clearance, potentially through direct effects on neutrophil function. In line with the Corriden et al.^14^ study, which suggests that e-cigarette use may impair immune function, our study found a significant reduction in circulating white blood cells (WBCs) and lymphocytes in the e-cig Nic+ group. This decline may be attributed to several mechanisms, including nicotine-induced immunosuppression, oxidative stress, and inflammatory responses. Nicotine has been shown to modulate immune function by activating nicotinic acetylcholine receptors (nAChRs) on immune cells, leading to impaired lymphocyte proliferation and function^15^. Additionally, oxidative stress generated by e-cigarette vapor can trigger apoptosis in immune cells, further contributing to the reduction in WBCs^7^. Chronic exposure may also alter bone marrow hematopoiesis, reducing leukocyte production and weakening immune defense^16^. Overall, the findings suggest that e-cigarette exposure induces inflammation and immune dysfunction. Several factors may explain the discrepancies between our study and others regarding the impact of e-cigarettes on oxidative stress and hematological parameters. These include variations in the duration of exposure across different species, experimental conditions, and differences between *in vitro* and *in vivo* models. Exploring these potential explanations could offer valuable insights into the mechanisms underlying e-cigarette-induced inflammatory responses.

Espinoza-Derout et al.^17^ exposed 8-week-old male C57BL/6J ApoE-/- mice to saline, e-cigarette aerosol without nicotine, or e-cigarette aerosol containing 2.4% nicotine for 12 weeks to investigate the impact of e-cigarette-exposure on inflammation-related gene expression in cardiac tissue. Transcriptomic analysis was performed to assess gene expression changes. The results demonstrated that e-cigarette exposure, particularly with nicotine, induced significant alterations in inflammation-related genes, characterized by the upregulation of pro-inflammatory genes and the downregulation of anti-inflammatory genes, indicating a shift towards a pro-inflammatory state. These findings suggest that chronic intermittent exposure to e-cigarette aerosols, especially those containing nicotine, can modulate inflammation-related gene expression, potentially contributing to cardiac dysfunction and atherosclerosis in this mouse model. In contrast, our study demonstrates that e-cigarette aerosol exposure downregulates the expression of 70 inflammatory genes in the post-MI heart compared to air-exposed control rats, including members of the TNF superfamily, interleukin family, and chemokine family. The discrepancy between the Espinoza-Derout et al.^17^ study and ours remains unclear; it may stem from differences in species or heart conditions and warrants further investigation. The suppression of inflammatory genes from our study raises critical questions about the long-term effects of e-cigarette use on the post-MI remodeling. While inflammation is a key driver of cardiovascular diseases, its complete suppression may also have negative consequences, such as impaired immune responses and delayed tissue repair. After MI, necrotic cardiomyocytes release danger signals that activate innate immune pathways, triggering an inflammatory response. Leukocytes infiltrate the infarcted tissue to clear dead cells, followed by anti-inflammatory signals that promote tissue repair. Fibroblasts proliferate and deposit extracellular matrix, leading to scar formation and maintaining structural integrity. However, dysregulated inflammation can cause adverse remodeling, increasing the risk of heart failure^18^. Our study suggests that future research should consider the long-term effects (beyond 12 weeks) of e-cigarette exposure on post-MI remodeling.

Platelets play a crucial role in vascular homeostasis, hemostasis, and immune responses, and their function can be significantly impacted by vaping. Hom et al.^19^ collected platelets from 50 healthy volunteers, exposed the platelets to tobacco smoke extracts, e-cigarette vapor extracts, and nicotine, assessing activation, adhesion, aggregation, and inflammation via optical aggregation, flow cytometry, and ELISA. This study found that e-cigarette vapor extracts significantly increased platelet activation, aggregation, and adhesion, as well as pro-inflammatory marker expression, with non-nicotine components playing a key role, although nicotine in extracts contributed to platelet functional changes in a dose-dependent manner. Qasim et al.^20^ investigated the effects of e-cigarette exposure on platelet function and thrombogenesis using a mouse model that mimics real-life human exposure. C57BL/6 mice were exposed to e-cigarette vapor (18 mg/mL nicotine content) for 5 days using a passive inhalation system mimicking real-life conditions (200 puffs/day, 3-second puffs, 1-minute intervals), while control mice were exposed to clean air, with experiments conducted one hour after the final exposure. Findings revealed that e-cigarette exposure induces platelet hyperactivity, enhances thrombogenic potential, and disrupts normal hemostasis, suggesting an increased risk of cardiovascular events and underscoring the need for further research on e-cigarette health risks. However, in our present study, a significant decrease in platelet count was detected in the e-cig Nic+ group compared to the air group after 12 weeks of exposure during the post-MI healing phase in rats. The underlying mechanisms remain unknown and require further investigation. Future studies should explore whether prolonged e-cigarette exposure induces bone marrow suppression or alters megakaryopoiesis, leading to reduced platelet production. Additionally, the role of oxidative stress, systemic inflammation, and endothelial dysfunction in platelet depletion should be examined. Investigating the potential impact of e-cigarette exposure on platelet lifespan, clearance, and apoptosis may provide further insights into the observed decrease in platelet count. Understanding these mechanisms could help clarify the long-term cardiovascular risks associated with chronic e-cigarette use, particularly in individuals with pre-existing cardiovascular conditions.

### Limitations

This study has several limitations. First, the health effects of e-cigarettes remain controversial, with some studies highlighting their adverse effects while others suggest they are safer alternatives to traditional tobacco products. This study lacks a tobacco smoke control group and a nicotine-free e-cigarette group, which would provide a more comprehensive comparison. Second, PCR analysis of inflammatory gene expression was conducted only in female rats, necessitating further validation in male rats to assess potential sex differences. Third, nicotine and cotinine plasma levels were not measured, which limits the ability to directly correlate exposure with physiological effects. Fourth, while the study aimed to investigate chronic exposure, the 12-week duration may not fully capture the long-term effects, as the model represents a more acute exposure rather than a truly chronic system. Fifth, potential sex differences in responses to e-cigarette exposure remain unexplored. Lastly, the use of young animals may limit the generalizability of findings, as age-related differences in susceptibility to e-cigarette exposure and myocardial infarction recovery should be considered in future studies.

## CONCLUSIONS

Chronic exposure to e-cigarette with nicotine (e-cig Nic+) vapor induces lung inflammation, triggers oxidative stress through ROS, alters cardiac inflammatory gene expression, and affects blood parameters during the healing phase of myocardial infarction in a rat model. Our findings suggest that chronic inhalation of e-cigarette aerosols contributes to lung lesions and suppresses immune and inflammatory responses in the already compromised MI setting.

## Supplementary Material



## Data Availability

The data supporting this research are available from the authors on reasonable request.
