# Supplementary material for: Inflammatory response to chronic nicotine-containing electronic cigarette exposure in a rat model of myocardial infarction
Source: Tob Induc Dis. 2025 May 31;23:10.18332/tid/204010. doi: 10.18332/tid/204010 (PMC12125711; doi:10.18332/tid/204010)
Supplement: Supplementary file 1 [file TID-23-74-s1.pdf]

RT² Profiler PCR Array Gene Expression Analysis Report

12-13-2022

Table of Contents

Introduction 3

Summary and workflow 4

Gene Table 5

Data analysis setup 8

Data quality control (QC) 9

Normalization analysis 10

Result 11

- Fold regulation and p-value 11
- Scatter Plot 12
- Volcano Plot 15
- Clustergram 18
- Heat Map 19

What's next 20

- Gene Expression, Protein Detection 21
- Transcription Factor / Histone 22

Introduction

Cataloged arrays

RT² Profiler PCR Arrays are highly reliable and sensitive gene expression profiling tools for analyzing focused panels of genes insignal transduction, biological processes or disease research pathways using real-time PCR. Each cataloged RT² Profiler PCR Array contains a list of the pathway-focused genes as well as five housekeeping (reference) genes on the array. In addition, eacharray contains a panel of proprietary controls to monitor genomic DNA contamination (GDC) as well as the first strand synthesis (RTC) and real-time PCR efficiency (PPC). The qPCR Assays used in PCR Arrays are laboratory-verified and optimized to workunder standard conditions enabling a large number of genes to be assayed simultaneously. Their specificity is guaranteed when RT² SYBR Green qPCR Master Mixes are used as part of the complete PCR Array System protocol.

In this study, 96 genes were profiled on 8 samples with the PARN-011Z.

Summary and workflow

Cataloged arrays

1. Mature RNA was isolated using an RNA extraction kit according to the manufacturer's instructions.
2. RNA quality was determined using a spectrophotometer and was reverse transcribed using a cDNA conversion kit.
3. The cDNA was used on the real-time RT^2 Profiler PCR Array (QIAGEN, Cat. no. PARN-011Z) in combination with RT^2 SYBR® Green qPCR Mastermix (Cat. no. 330529).

C_T values were exported to an Excel file to create a table of C_T values. This table was then uploaded on to the data analysis web portal at http://www.qiagen.com/geneglobe. Samples were assigned to controls and test groups. C_T values were normalized based on a/an Automatic Selection from HKG Panel of reference genes.

The data analysis web portal calculates fold change/regulation using delta delta C_T method, in which delta C_T is calculated between gene of interest (GOI) and an average of reference genes (HKG), followed by delta-delta C_T calculations (delta C_T (Test Group)-delta C_T (Control Group)). Fold Change is then calculated using 2 ^ (-delta delta C_T) formula. The data analysis web portal also plots scatter plot, volcano plot, clustergram, and heat map.

This data analysis report was exported from the QIAGEN web portal at GeneGlobe.

Gene Table

Position	RefSeq Number	Symbol	Description
A01	NM_053757	Aimp1	Aminoacyl tRNA synthetase complex-interacting multifunctional protein 1
A02	NM_017178	Bmp2	Bone morphogenetic protein 2
A03	NM_019205	Ccl11	Chemokine (C-C motif) ligand 11
A04	NM_001105822	Ccl12	Chemokine (C-C motif) ligand 12
A05	NM_057151	Ccl17	Chemokine (C-C motif) ligand 17
A06	NM_001108661	Ccl19	Chemokine (C-C motif) ligand 19
A07	NM_031530	Ccl2	Chemokine (C-C motif) ligand 2
A08	NM_019233	Ccl20	Chemokine (C-C motif) ligand 20
A09	NM_057203	Ccl22	Chemokine (C-C motif) ligand 22
A10	NM_001013045	Ccl24	Chemokine (C-C motif) ligand 24
A11	NM_013025	Ccl3	Chemokine (C-C motif) ligand 3
A12	NM_053858	Ccl4	Chemokine (C-C motif) ligand 4
B01	NM_031116	Ccl5	Chemokine (C-C motif) ligand 5
B02	NM_001004202	Ccl6	Chemokine (C-C motif) ligand 6
B03	NM_001007612	Ccl7	Chemokine (C-C motif) ligand 7
B04	NM_001012357	Ccl9	Chemokine (C-C motif) ligand 9
B05	NM_020542	Ccr1	Chemokine (C-C motif) receptor 1
B06	NM_001108836	Ccr10	Chemokine (C-C motif) receptor 10
B07	NM_021866	Ccr2	Chemokine (C-C motif) receptor 2
B08	NM_053958	Ccr3	Chemokine (C-C motif) receptor 3
B09	NM_133532	Ccr4	Chemokine (C-C motif) receptor 4
B10	NM_053960	Ccr5	Chemokine (C-C motif) receptor 5
B11	NM_001013145	Ccr6	Chemokine (C-C motif) receptor 6
B12	XM_008757885	Ccr8	Chemokine (C-C motif) receptor 8
C01	NM_053353	Cd40lg	CD40 ligand
C02	NM_023981	Csf1	Colony stimulating factor 1 (macrophage)
C03	NM_053852	Csf2	Colony stimulating factor 2 (granulocyte-macrophage)
C04	NM_017104	Csf3	Colony stimulating factor 3 (granulocyte)
C05	NM_134455	Cx3cl1	Chemokine (C-X3-C motif) ligand 1
C06	NM_133534	Cx3cr1	Chemokine (C-X3-C motif) receptor 1
C07	NM_030845	Cxcl1	Chemokine (C-X-C motif) ligand 1 (melanoma growth stimulating activity, alpha)
C08	NM_139089	Cxcl10	Chemokine (C-X-C motif) ligand 10
C09	NM_182952	Cxcl11	Chemokine (C-X-C motif) ligand 11
C10	NM_022177	Cxcl12	Chemokine (C-X-C motif) ligand 12 (stromal cell-derived factor 1)

Position	RefSeq Number	Symbol	Description
C11	NM_053647	Cxcl2	Chemokine (C-X-C motif) ligand 2
C12	NM_022214	Cxcl6	Chemokine (C-X-C motif) ligand 5
D01	NM_145672	Cxcl9	Chemokine (C-X-C motif) ligand 9
D02	NM_017183	Cxcr2	Chemokine (C-X-C motif) receptor 2
D03	NM_053415	Cxcr3	Chemokine (C-X-C motif) receptor 3
D04	NM_053303	Cxcr5	Chemokine (C-X-C motif) receptor 5
D05	NM_012908	Faslg	Fas ligand (TNF superfamily, member 6)
D06	NM_138880	Ifng	Interferon gamma
D07	NM_057193	Il10ra	Interleukin 10 receptor, alpha
D08	NM_133519	Il11	Interleukin 11
D09	NM_053828	Il13	Interleukin 13
D10	NM_013129	Il15	Interleukin 15
D11	NM_001105749	Il16	Interleukin 16
D12	NM_001106897	Il17a	Interleukin 17A
E01	NM_053789	Il17b	Interleukin 17B
E02	NM_001015011	Il17f	Interleukin 17F
E03	NM_017019	Il1a	Interleukin 1 alpha
E04	NM_031512	Il1b	Interleukin 1 beta
E05	NM_013123	Il1r1	Interleukin 1 receptor, type I
E06	NM_022194	Il1rn	Interleukin 1 receptor antagonist
E07	NM_001108943	Il21	Interleukin 21
E08	XM_344962	Il27	Interleukin 27
E09	NM_013195	Il2rb	Interleukin 2 receptor, beta
E10	NM_080889	Il2rg	Interleukin 2 receptor, gamma
E11	NM_031513	Il3	Interleukin 3
E12	NM_001014166	Il33	Interleukin 33
F01	NM_201270	Il4	Interleukin 4
F02	NM_021834	Il5	Interleukin 5
F03	NM_053645	Il5ra	Interleukin 5 receptor, alpha
F04	NM_017020	Il6r	Interleukin 6 receptor
F05	NM_001008725	Il6st	Interleukin 6 signal transducer
F06	NM_013110	Il7	Interleukin 7
F07	NM_019310	Cxcr1	Interleukin 8 receptor, alpha
F08	NM_080769	Lta	Lymphotoxin alpha (TNF superfamily, member 1)
F09	NM_212507	Ltb	Lymphotoxin beta (TNF superfamily, member 3)
F10	NM_031051	Mif	Macrophage migration inhibitory factor
F11	NM_177928	Nampt	Nicotinamide phosphoribosyltransferase

Position	RefSeq Number	Symbol	Description
F12	NM_001006961	Osm	Oncostatin M
G01	NM_001007729	Pf4	Platelet factor 4
G02	XM_001079130	RGD1561905_predicted	Complement component 5
G03	NM_012881	Spp1	Secreted phosphoprotein 1
G04	NM_012675	Tnf	Tumor necrosis factor (TNF superfamily, member 2)
G05	NM_012870	Tnfrsf11b	Tumor necrosis factor receptor superfamily, member 11b
G06	NM_145681	Tnfsf10	Tumor necrosis factor (ligand) superfamily, member 10
G07	NM_057149	Tnfsf11	Tumor necrosis factor (ligand) superfamily, member 11
G08	NM_001009623	Tnfsf13	Tumor necrosis factor (ligand) superfamily, member 13
G09	NM_001109112	Tnfsf13b	Tumor necrosis factor (ligand) superfamily, member 13b
G10	NM_001191803	Tnfsf14	Tumor necrosis factor (ligand) superfamily, member 14
G11	NM_053552	Tnfsf4	Tumor necrosis factor (ligand) superfamily, member 4
G12	NM_031836	Vegfa	Vascular endothelial growth factor A
H01	NM_031144	Actb	Actin, beta
H02	NM_012512	B2m	Beta-2 microglobulin
H03	NM_012583	Hprt1	Hypoxanthine phosphoribosyltransferase 1
H04	NM_017025	Ldha	Lactate dehydrogenase A
H05	NM_001007604	Rplp1	Ribosomal protein, large, P1
H06	U26919	RGDC	Rat Genomic DNA Contamination
H07	SA_00104	RTC	Reverse Transcription Control
H08	SA_00104	RTC	Reverse Transcription Control
H09	SA_00104	RTC	Reverse Transcription Control
H10	SA_00103	PPC	Positive PCR Control
H11	SA_00103	PPC	Positive PCR Control
H12	SA_00103	PPC	Positive PCR Control

Data analysis setup

Sample management

Sample ID	Sample Name	Group
1	MI/Air	Control Group
2	MI/Air.1	Control Group
3	MI/Air.2	Control Group
4	MI/Air.3	Control Group
5	MI/eC NIC+	Group 1
6	MI/eC NIC+.1	Group 1
7	MI/eC NIC+.2	Group 1
8	MI/eC NIC+.3	Group 1

Pre-amplification

A pre-amplification using the appropriate species- and pathway-specific RT^2 PreAMP Primer Mix was not performed and no corrections were made to C_T values during the data analysis procedure other than the use of the C_T cut-off value.

Lower limit of detection

The C_T cut-off was set to 35

Data quality control (QC)

Quality checks performed and results

Test Performed	Test Result
1. PCR Array Reproducibility	All Samples Passed
2. Reverse Transcription Efficiency	All Samples Passed
3. Genomic DNA Contamination	All Samples Passed

Normalization analysis

Automatic Selection from HKG Panel

Groups	Samples	Actb	Geometric Mean	Average Geometric Mean
Control Group	MI/Air	20.19	20.19	20.63
Control Group	MI/Air.1	20.52	20.52	
Control Group	MI/Air.2	20.75	20.75	
Control Group	MI/Air.3	21.05	21.05	
Group 1	MI/eC NIC+	20.55	20.55	20.54
Group 1	MI/eC NIC+.1	20.44	20.44	
Group 1	MI/eC NIC+.2	20.51	20.51	
Group 1	MI/eC NIC+.3	20.67	20.67	

In the Automatic Selection from HKG Panel method, the software automatically selected the listed optimal set of housekeeping / reference genes with the most stable expression across the Samples based on the results of the PCR Array's housekeeping / reference gene set. The geometric mean of the genes' assays' data was used as the normalization factor.

Result

Fold regulation and p-value

Test Group	Control Group	Fold Regulation Threshold	p-Value Threshold
Group 1	Control Group	1.5	0.05

Position	Gene Symbol	Fold Regulation	p-Value	Comments
A04	Ccl12	-2.71	0.014588	A
A05	Ccl17	-2.61	0.018042	
A10	Ccl24	-2.28	0.025111	
A11	Ccl3	-2.66	0.018190	A
B01	Ccl5	-1.55	0.027375	
C01	Cd40lg	-1.91	0.047266	
D03	Cxcr3	-2.28	0.006680	
D05	Faslg	-1.65	0.018881	A
E04	Il1b	-6.49	0.004706	A
G04	Tnf	-2.63	0.022664	
G10	Tnfsf14	-2.63	0.021499	
H03	Hprt1	-2.42	0.049243	

Fold-Change (2 ^ (-Delta Delta C_T)) is the normalized gene expression (2 ^ (- Delta C_T)) in the Test Sample divided the normalized gene expression (2 ^ (- Delta C_T)) in the Control Sample. Fold-Regulation represents fold-change results in a biologically meaningful way. Fold-change values greater than one indicates a positive- or an up-regulation, and the fold-regulation is equal to the fold-change. Fold-change values less than one indicate a negative or down-regulation, and the fold-regulation is the negative inverse of the fold-change.

The p values are calculated based on a Student's t-test of the replicate 2 ^ (- Delta C_T) values for each gene in the control group and treatment groups, and p values less than 0.05 are indicated in red. The p-value calculation used is based on parametric, unpaired, two-sample equal variance, two-tailed distribution – a method widely accepted in scientific literature.

Scatter Plot

Test Group	Control Group	Fold Regulation Threshold
Group 1	Control Group	1.5

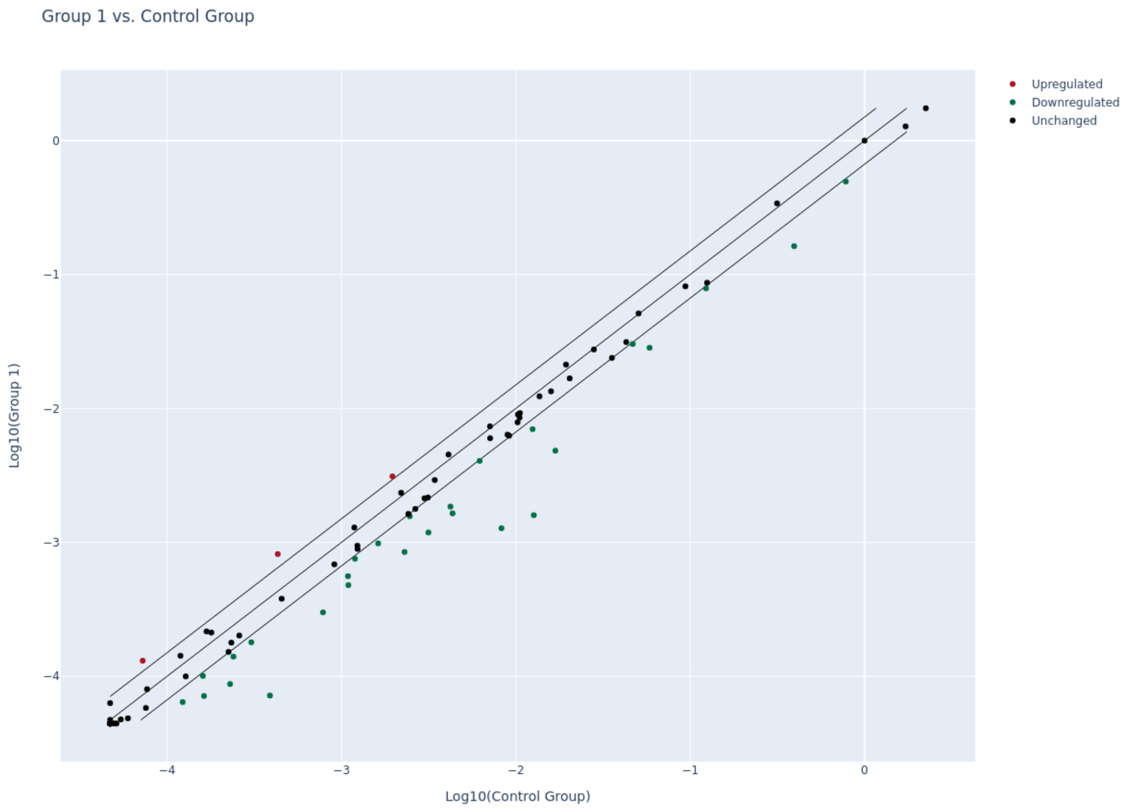

The Scatter Plot compares the normalized expression of every gene on the PCR Array between the two selected groups by plotting them against one another to quickly visualize large gene expression changes. The center diagonal line indicates unchanged gene expression, while the outer diagonal lines indicate the selected fold regulation threshold. Genes with data points beyond the outer lines in the upper left and lower right corners are up-regulated or down-regulated, respectively, by more than the fold regulation threshold in the y-axis Group relative to the x-axis Group.

Genes Over-Expressed in Group 1 vs. Control Group

Position	Gene Symbol	Fold Regulation	Comments	RT² qPCR Assay Catalog #
D04	Cxcr5	1.90	B	PPR06524A
E08	Il27	1.80	B	PPR63375F
G05	Tnfrsf11b	1.59		PPR06478A

Genes Under-Expressed in Group 1 vs. Control Group

Position	Gene Symbol	Fold Regulation	Comments	RT² qPCR Assay Catalog #
A02	Bmp2	-1.57		PPR06531B
A04	Ccl12	-2.71	A	PPR52425A
A05	Ccl17	-2.61		PPR06445A
A07	Ccl2	-1.78		PPR06714B
A08	Ccl20	-1.71	B	PPR06386A
A09	Ccl22	-1.60	B	PPR06451A
A10	Ccl24	-2.28		PPR50989B
A11	Ccl3	-2.66	A	PPR06717A
B01	Ccl5	-1.55		PPR06854F
B06	Ccr10	-1.58	B	PPR50407A
B12	Ccr8	-2.28	B	PPR57707A
C01	Cd40lg	-1.91		PPR49715A
C07	Cxcl1	-7.94		PPR06663A
C09	Cxcl11	-3.48		PPR45861C
C11	Cxcl2	-5.42	B	PPR06720B
D03	Cxcr3	-2.28		PPR06418A
D05	Faslg	-1.65	A	PPR06476A
D06	Ifng	-1.69	B	PPR45050C
E03	Il1a	-1.95	B	PPR06403C
E04	Il1b	-6.49	A	PPR06480B
E09	Il2rb	-1.53		PPR06454F
F10	Mif	-1.56		PPR42812B
G01	Pf4	-2.06		PPR06698A
G04	Tnf	-2.63		PPR06411F
G10	Tnfsf14	-2.63		PPR57765A
H03	Hprt1	-2.42		PPR42247F
H04	Ldha	-1.58		PPR56603B

Volcano Plot

Test Group	Control Group	Fold Regulation Threshold	p-Value Threshold
Group 1	Control Group	1.5	0.05

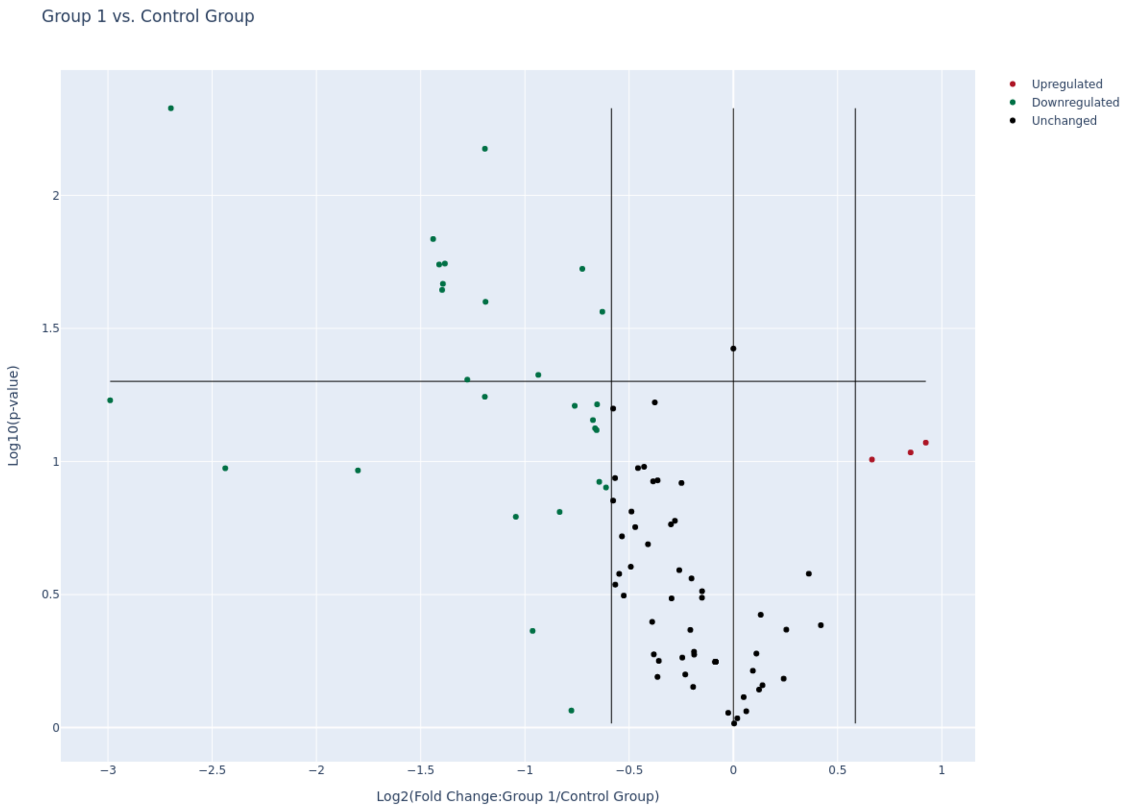

The Volcano Plot identifies significant gene expression changes by plotting the log2 of the fold changes in gene expression on the x-axis versus their statistical significance on the y-axis. The center vertical line indicates unchanged gene expression, while the two outer vertical lines indicate the selected fold regulation threshold. The horizontal line indicates the selected p-value threshold. Genes with data points in the far upper left (down-regulated) and far upper right (up-regulated) sections meet the selected fold regulation and p-value thresholds. By combining the fold change results with the p-value statistical test results, genes with both large and small expression changes that are statistically significant are easily visualized.

Genes Over-Expressed in Group 1 vs. Control Group

Position	Gene Symbol	Fold Regulation	p-Value	Comments	RT² qPCR Assay Catalog #
D04	Cxcr5	1.90	0.084955	B	PPR06524A
E08	Il27	1.80	0.092466	B	PPR63375F
G05	Tnfrsf11b	1.59	0.098316		PPR06478A

Genes Under-Expressed in Group 1 vs. Control Group

Position	Gene Symbol	Fold Regulation	p-Value	Comments	RT² qPCR Assay Catalog #
A02	Bmp2	-1.57	0.060995		PPR06531B
A04	Ccl12	-2.71	0.014588	A	PPR52425A
A05	Ccl17	-2.61	0.018042		PPR06445A
A07	Ccl2	-1.78	0.154711		PPR06714B
A08	Ccl20	-1.71	0.862291	B	PPR06386A
A09	Ccl22	-1.60	0.069867	B	PPR06451A
A10	Ccl24	-2.28	0.025111		PPR50989B
A11	Ccl3	-2.66	0.018190	A	PPR06717A
B01	Ccl5	-1.55	0.027375		PPR06854F
B06	Ccr10	-1.58	0.075042	B	PPR50407A
B12	Ccr8	-2.28	0.057117	B	PPR57707A
C01	Cd40lg	-1.91	0.047266		PPR49715A
C07	Cxcl1	-7.94	0.058892		PPR06663A
C09	Cxcl11	-3.48	0.108023		PPR45861C
C11	Cxcl2	-5.42	0.105996	B	PPR06720B
D03	Cxcr3	-2.28	0.006680		PPR06418A
D05	Faslg	-1.65	0.018881	A	PPR06476A
D06	Ifng	-1.69	0.061766	B	PPR45050C
E03	Il1a	-1.95	0.432960	B	PPR06403C
E04	Il1b	-6.49	0.004706	A	PPR06480B
E09	Il2rb	-1.53	0.125169		PPR06454F
F10	Mif	-1.56	0.119253		PPR42812B
G01	Pf4	-2.06	0.161288		PPR06698A
G04	Tnf	-2.63	0.022664		PPR06411F
G10	Tnfsf14	-2.63	0.021499		PPR57765A
H03	Hprt1	-2.42	0.049243		PPR42247F
H04	Ldha	-1.58	0.076222		PPR56603B

Clustergram

Sample	Dimension	Join Type	Color Coded
Array	2-D	Average	Genes

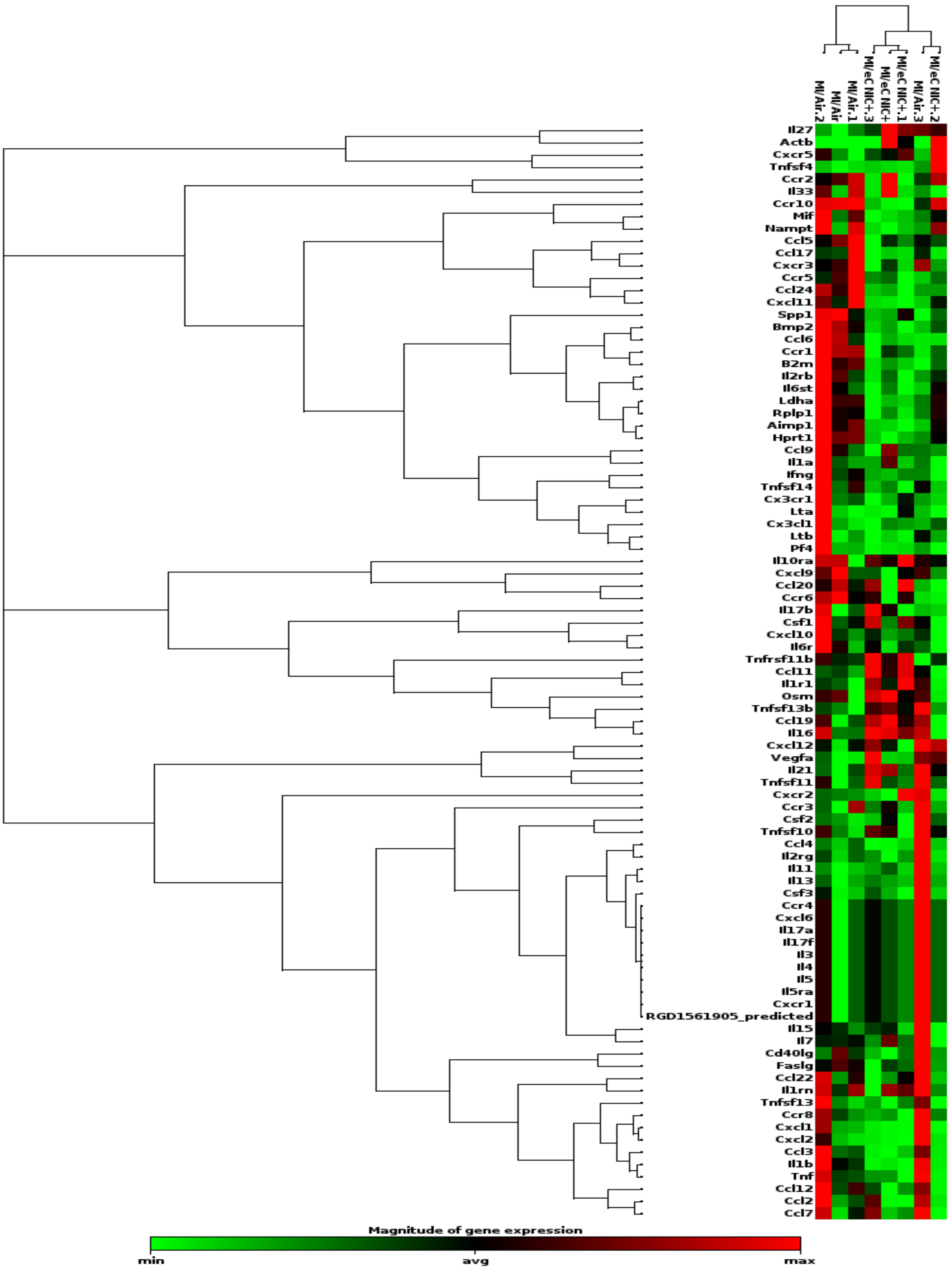

Heat Map

Test Group	Control Group
Group 1	Control Group

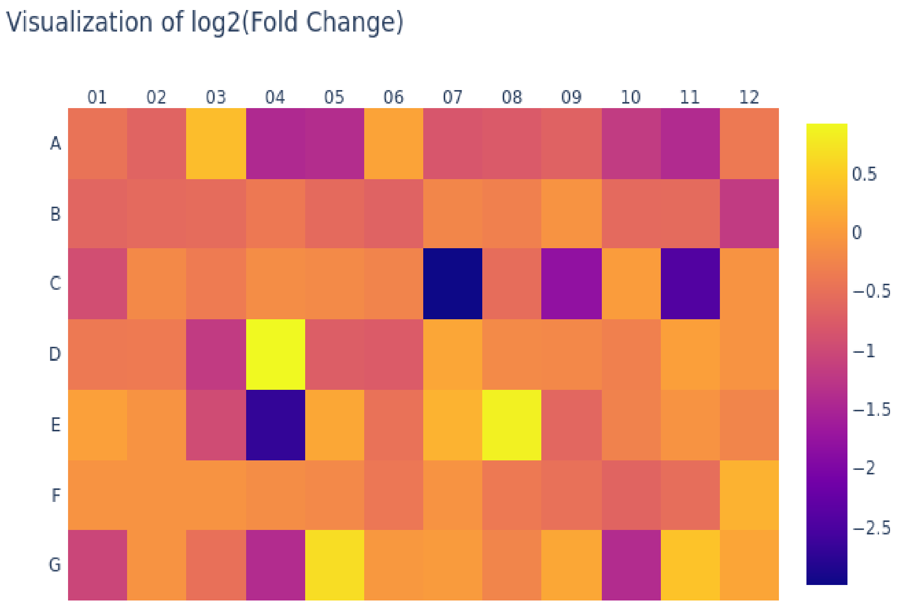

Layout	01	02	03	04	05	06	07	08	09	10	11	12
A	Aimp1 / -1.37	Bmp2 / -1.57	Ccl11 / 1.28 / B	Ccl12 / -2.71 / A	Ccl17 / -2.61	Ccl19 / 1.07	Ccl2 / -1.78	Ccl20 / -1.71 / B	Ccl22 / -1.6 / B	Ccl24 / -2.28	Ccl3 / -2.66 / A	Ccl4 / -1.31 / B
B	Ccl5 / -1.55	Ccl6 / -1.49	Ccl7 / -1.46	Ccl9 / -1.31 / B	Ccr1 / -1.48	Ccr10 / -1.58 / B	Ccr2 / -1.17	Ccr3 / -1.23 / B	Ccr4 / -1.06 / C	Ccr5 / -1.49	Ccr6 / -1.48 / B	Ccr8 / -2.28 / B
C	Cd40lg / -1.91	Csf1 / -1.14	Csf2 / -1.28 / B	Csf3 / -1.11 / B	Cx3cl1 / -1.14	Cx3cr1 / -1.2 / B	Cxcl1 / -7.94	Cxcl10 / -1.45	Cxcl11 / -3.48	Cxcl12 / 1.01	Cxcl2 / -5.42 / B	Cxcl6 / -1.06 / C
D	Cxcl9 / -1.3	Cxcr2 / -1.29 / B	Cxcr3 / -2.28	Cxcr5 / 1.9 / B	Faslg / -1.65 / A	Ifng / -1.69 / B	Il10ra / 1.09 / B	Il11 / -1.14 / B	Il13 / -1.15 / B	Il15 / -1.23	Il16 / 1.03	Il17a / -1.06 / C
E	Il17b / 1.04 / B	Il17f / -1.06 / C	Il1a / -1.95 / B	Il1b / -6.49 / A	Il1r1 / 1.1	Il1rn / -1.39 / B	Il21 / 1.19 / B	Il27 / 1.8 / B	Il2rb / -1.53	Il2rg / -1.21	Il3 / -1.06 / C	Il33 / -1.19
F	Il4 / -1.06 / C	Il5 / -1.06 / C	Il5ra / -1.06 / C	Il6r / -1.11	Il6st / -1.15	Il7 / -1.33 / B	Cxcr1 / -1.06 / C	Lta / -1.3 / B	Ltb / -1.4	Mif / -1.56	Nampt / -1.44	Osm / 1.18 / B
G	Pf4 / -2.06	RGD1561905_predicted / -1.06 / C	Spp1 / -1.41	Tnf / -2.63	Tnfrsf11b / 1.59	Tnfsf10 / -1.02	Tnfsf11 / 1.0 / B	Tnfsf13 / -1.19	Tnfsf13b / 1.1	Tnfsf14 / -2.63	Tnfsf4 / 1.34 / B	Vegfa / 1.08

What's next

Thank you for using the RT^2 Profiler Data Analysis Software.

The Data Analysis software delivers a list of expression changes in the samples from the supplied data. However, this result often only starts an investigation into the underlying mechanisms at work. In order to assist in further analysis, the QIAGEN now utilizes the latest bioinformatics tools to analyze the data and suggest regulatory mechanisms and future experiments. Please review the results from the selected tools below.

Gene Expression: This tool will help define a panel of genes based of this experiment's results. This panel may represent aputative biomarker set, a target gene set or simply a collection of genes. The tool is designed to deliver a list of gene expressionassays that would allow the user to follow-up the results of the analyzed experiment.

Transcription Factor / Histone: This tool will help define a panel of differentially expressed genes based on this experiment's results. Altered transcription factor binding activity on the genes' promoters may be responsible for these gene expression changes. Altered histone modification patterns on the genes' promoters may also be responsible for these gene expression changes. This tool is designed to deliver a list of the transcription factors that might regulate the selected differentially expressed genes as well as the available respective gene-specific real-time PCR assays for DNA from anti-transcription factor oranti-histone chromatin immunoprecipitations. These assays would then allow the user to follow-up their gene expression experiment with an epigenetic analysis.

Protein Detection:This tool will help define a panel of cytokines or chemokines based on this experiment's results. This panel may represent a putative biomarker set, a target gene set or simply a collection of genes. The tool is designed to deliver a list of ELISAs that would allow the user to follow-up the results of the analyzed experiment.

Gene Expression, Protein Detection

Test Group	Control Group	Fold Regulation Threshold	p-Value Threshold
Group 1	Control Group	1.5	0.05

Position	Symbol	Fold Regulation	p-Value	RT2 qPCR Assay	Single Analyte ELISArray
A04	Ccl12	-2.71	0.014588	PPR52425A	
A05	Ccl17	-2.61	0.018042	PPR06445A	
A10	Ccl24	-2.28	0.025111	PPR50989B	
A11	Ccl3	-2.66	0.01819	PPR06717A	
B01	Ccl5	-1.55	0.027375	PPR06854F	SER06854A
C01	Cd40lg	-1.91	0.047266	PPR49715A	
D03	Cxcr3	-2.28	0.00668	PPR06418A	
D05	Faslg	-1.65	0.018881	PPR06476A	
E04	Il1b	-6.49	0.004706	PPR06480B	
G04	Tnf	-2.63	0.022664	PPR06411F	SER06411A
G10	Tnfsf14	-2.63	0.021499	PPR57765A	
H03	Hprt1	-2.42	0.049243	PPR42247F	

Transcription Factor / Histone

Test Group	Control Group	Fold Regulation Threshold	p-Value Threshold
Group 1	Control Group	1.5	0.05

Genes Differentially Expressed in Group 1 vs. Control Group

Position	Gene Symbol	Fold Regulation	p-Value	EpiTect ChIP qPCR Assay	Transcription Factors
A04	Ccl12	-2.71	0.014588	GPR1058078(-)01A	TBP, TFIID
A05	Ccl17	-2.61	0.018042	GPR1072573(-)01A	FOXJ2 (long isoform), FOXJ2, RORalpha1, STAT5B, RORalpha2, C/EBPalpha
A10	Ccl24	-2.28	0.025111	GPR1058869(-)01A	AP-1, TBP
A11	Ccl3	-2.66	0.01819	GPR1069498(-)01A	IRF-7A, C/EBPalpha, FAC1, RSRFC4, Hlf, C/EBPbeta, TFIID, HOXA9, TBP, aMEF-2, MEF-2A, HOXA9B, Meis-1, AML1a, Meis-1b
B01	Ccl5	-1.55	0.027375	GPR1069495(-)01A	NF-kappaB2, IRF-1, NF-kappaB, c-Rel, IRF-2, TFIID, ER-alpha, RelA, TBP, ISGF-3, NF-kappaB1
C01	Cd40lg	-1.91	0.047266	GPR1067397(-)01A	IRF-7A, Nkx3-1 v2, IRF-1, Nkx3-1 v1, IRF-2, S8, STAT1, Sox9, Nkx3-1, Nkx3-1 v3, FOXO3, POU3F2 (N-Oct-5b), STAT2, STAT5B, Meis-1, Nkx3-1 v4, FOXO3b, STAT6, POU3F2 (N-Oct-5a), PPAR-gamma1, STAT5A, STAT1alpha, Hlf, ATF-2, STAT1beta, HOXA9, PPAR-gamma2, FOXO3a, HOXA9B, STAT4, STAT3, POU3F2, Meis-1a
D03	Cxcr3	-2.28	0.00668	GPR1078735(-)01A	nan
D05	Faslg	-1.65	0.018881	GPR1070667(-)01A	Egr-3, Egr-4, Egr-2
E04	Il1b	-6.49	0.004706	GPR1074500(-)01A	NF-AT3, NF-AT, c-Rel, C/EBPbeta, TFIID, NF-AT1, RelA, TBP, NF-AT2, NF-AT4
G04	Tnf	-2.63	0.022664	GPR1062267(-)01A	CP1A, Egr-4, NF-YB, En-1, NF-kappaB, CBF-C, NF-Y, RelA, CBF-A, NF-YC, Egr-1, CP1C, NF-kappaB1, CBF-B, CBF(2), NF-YA
G10	Tnfsf14	-2.63	0.021499	nan	nan
H03	Hprt1	-2.42	0.049243	GPR1067375(-)01A	nan

© 2025 Shi J. et al.
